# A Novel Technique for Fiber Formation: Mechanotropic Spinning—Principle and Realization

**DOI:** 10.3390/polym10080856

**Published:** 2018-08-02

**Authors:** Valery G. Kulichikhin, Ivan Yu. Skvortsov, Andrey V. Subbotin, Sergey V. Kotomin, Alexander Ya. Malkin

**Affiliations:** A.V. Topchiev Institute of Petrochemical Synthesis, Russian Academy of Sciences 29, Leninsky prospect, Moscow 119991, Russia; amber5@yandex.ru (I.Y.S.); subbotin@ips.ac.ru (A.V.S.); svk@ips.ac.ru (S.V.K.); Alex_malkin@mig.phys.msu.ru (A.Y.M.)

**Keywords:** polymer solutions, fiber spinning, polyacrylonitrile, aromatic polyamide, uniaxial extension, phase separation

## Abstract

We present basic experimental data and the theoretical background of a novel technique for fiber spinning from polymer solutions. The principal feature of the advanced process is realization of phase separation with detachment of a solvent, accompanied by the orientation of macromolecules, under the action of high extension rates. This is similar in some respects to dry spinning, though the driving force is not diffusion with subsequent evaporation of a solvent but redistribution of polymer-solvent interactions in favor of polymer-polymer and solvent-solvent ones governed by mechanical stresses. A promise of this approach has been demonstrated by experiments performed with polyacrylonitrile solutions in different solvents and solutions of the rigid-chain aromatic polyamide. We examined mechanotropic fiber spinning in model experiments with stretching jets from a drop of polymer solution in different conditions, and then demonstrated the possibility of realizing this process in the stable long-term continuous mode. During extension, phase separation happens throughout the whole section of a jet, as was confirmed by visual observation. Then a solvent diffuses on a jet surface, forming a liquid shell on the oriented fiber. Instability of this cover due to surface tension leads either to formation of separate solvent drops “seating” on the fiber or to the flow of a solvent down to the Taylor cone. The separate liquid droplets can be easily taken off a fiber. The physics underlying this process is related to the analysis of the influence of macromolecule coil-to-stretched chain transition on the intermolecular interaction.

## 1. Introduction

Extension of polymeric fluids is one of the fundamental modes of deformation in different technological processes. Approximately 60 million tons of polymers are spent on fiber processing compared with the common polymer production of 335 million tons per year (in 2017). This is a very significant part. Therefore, modeling of the uniaxial extension of polymers is the biggest challenge in theoretical and applied rheology, having the goal of understanding the physics and mechanics of extension as the basis for the technology.

The rheology of extension has a long prehistory starting from the classical Trouton law, which is based on the fundamental concepts of fluid mechanics. According to this law, the elongation viscosity of a viscous fluid is equal to 3η where η is shear viscosity determined by the classical Newton-Stokes linear hypothesis. However, this law is valid only if the flow is steady and purely viscous. Polymeric fluids are viscoelastic, and the modes of flow usually are very rarely steady. Therefore, correct estimation of the flow of polymeric fluid continues to be an issue which has been treated in hundreds of publications and causes an active discussion, numerous contradictory judgments and possible errors.

The physical origin of the possibility of large deformations in extension is due to the extensibility of flexible macromolecular chains, and thus to polymer elasticity. Therefore, the extension should be considered a superposition of elastic (recoverable) and irreversible (flow) deformations, and steady flow will be realized after storage of some elastic deformations [1,2,3]. It is also important that at high deformation rates, namely when the Weissenberg number exceeds one (Wi >> 1), elastic deformations become dominant and extension takes place via an increase of elastic deformation till rubber-like rupture [4,5,6,7,8,9,10]. This line of investigation was related primarily to polymer melts and tendency to reach a steady state regime of extension.

The method of uniaxial elongation of polymer melts has been widely used, bearing in mind two purposes: evaluation of non-linear constitutive models (e.g., [11,12,13,14,15,16]) and characterization of polymers differing in their molecular-weight-distribution and/or architecture of polymer chains, especially considering the strain hardening effect [17,18,19,20,21,22,23,24,25,26,27].

It is also important to keep in mind that extension at high draw ratios can be accompanied by instability and appearance of inhomogeneity along a specimen (in particular, necking) that might lead to an incorrect understanding of experimental results [28,29,30,31,32,33]. Therefore, the control of correspondence between local and integral parameters of the extension process is always necessary [34].

The other approach related mainly to polymer solutions, including dilute ones, was developed based on the study of transient regimes of extension. After the first publication [35] and its theoretical grounding [36], a lot of papers appeared in which viscosity and relaxation properties of dilute polymer solutions were measured by the method of the filament thinning under the action of capillary forces [37,38,39,40,41,42,43,44,45,46,47,48], and the possibility of using this method for dilute solutions is a serious advantage of this technique. A comprehensive review of the filament stretching rheometers in applications to polymer solutions showed that this method allows for a rather detailed analysis of the rheology of complex fluids [49].

An especially noteworthy feature of high rate stretching is stress-induced phase transitions in extension. Naturally, such transitions are absent if we deal with amorphous polymers, where only a flow-to-rubbery transition at a high Weissenberg number happens. However, extension of crystallized polymer melts can lead to stress-induced crystallization [49,50,51,52,53,54]. This effect has been explained by deformation-induced nucleation in a melt [55]. The possibility of nematic structure formation in extension has also been described [56].

In addition, the extension of polymer solutions can lead to appearance of heterogeneous structures. Such observations were usually treated as the formation of bead-on-string morphology [57,58] which is usually considered to be instability of viscoelastic jets [59,60,61]. However, in all these cases, a jet was assumed to be a unit object with macromolecular orientation different in a bead and a string. It was noted that the final stage of extension of dilute polymer solutions is accompanied by blistering [62,63].

The phenomenon of “phase separation” in stretching of dilute solutions was previously described for concentrated solutions [64]. However, there was no follow-up to this experimental observation at that time, though many publications demonstrating shear-induced phase separation in amorphous polymer solutions appeared [65,66,67,68]. Recently, the phenomenon of phase separation in stretching of polymer solutions was observed in some studies [69,70,71,72,73]. Extending of this approach on semi-diluted solutions without entanglements has been done in the Appendix A.

Therefore, the basic concept underlying this study is the experimental evidence of phase separation in the system polymer-solvent under the action of high deformation rate in the uniaxial extension. This allows for not using a large amount of precipitant which is necessary in standard “wet” or “dry-wet jet” fiber spinning. This phenomenon could be the basis of a novel technology of fiber formation, known as mechanotropic spinning, where the oriented fiber is obtained from a polymer solution as a result of solvent removal due to phase separation at a high deformation rate. This paper is devoted to the development of this technique in fiber spinning from solutions of different polymers. 

The closest to the here developed e technique is electrospinning. Indeed, both techniques are realized at high deformation rates and removal of a solvent occurs without using a coagulant/precipitant. However, we pay special attention to the phase separation as the fundamental element of mechanotropic spinning, while the main mechanism considered in electrospinning is evaporation of a solvent. In addition, generally (though not always), electrospinning is oriented to formation of non-woven mats while mechanotropic process is aimed at continuous winding of fibers. Nevertheless, it is reasonable to suppose that the basic physical mechanism of removal of solvent from polymer solutions is similar in both cases.

## 2. Materials and Methods

### 2.1. Samples

We conducted experiments with two types of fiber-forming polymers. The first one was polyacrylonitrile (PAN), produced by Good Fellow (Great Britain, trade mark AN316020). The PAN sample was characterized by the following parameters: composition—acrylonitrile/methyl acrylate/methyl sulfonate ratio equal to 93.5/5.8/0.3 wt. % and average molecular weight of 8.5 × 10^4^ g/mole, determined via intrinsic viscosity and M_w_/M_n_ = 1.92. Solutions of PAN in dimethyl sulfoxide (DMSO), dimethylacetamide (DMAc), dimethylformamide (DMF), and *N*-Methyl-2-pyrrolidone (NMP) were prepared. These solvents differ in affinity to PAN. It changes in accordance with the following rank: DMSO > DMF > DMAc~NMP [74]. All solvents were produced by Ecos-1 Co. (Russia) and were used as supplied. The concentration of the solution was 25 wt. %, because this concentration provides the best spinnability.

The second polymer was a rigid-chain aromatic copolymer of 5(6)amine-2(p-phenylamine)benzimidazole with terephthaloyl chloride and *p*-phenylenediamine (CPABI), a founder of one of the Russian versions of the Kevlar-type fibers—Armos. Its formula is shown below: 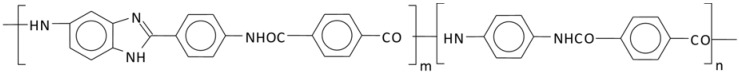


The intrinsic viscosity of the CPABI sample in DMAc (+3% LiCl) at 30 °C is equal to 5.95 dL/g, the number average molecular weight determined by GPC method is 42 × 10^3^ g/mole and the polydispersity index is close to 2.3. Concentrations of solutions under investigation in DMAc with LiCl were 7.0 and 9.9 wt. %. 

### 2.2. Experimental Setup

**Figure 1:**
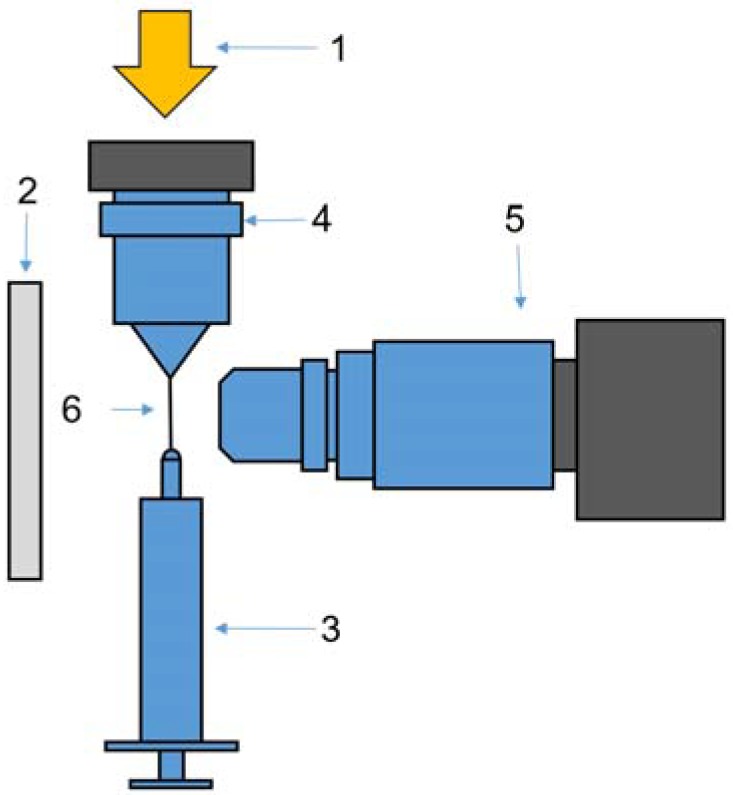
Experimental device for stretching a jet up to a definite length. 1—fiber-optic illuminator along the jet axis; 2—back lightening; 3—syringe with solution; 4—lens to focus the light into the center of an extended jet; 5—camera; 6—jet.

Two different experimental setups were used. The first one is a version of the model device on extension of a droplet, similar to that described in detail elsewhere [72]. This is a jet drawing from the drop of a polymer solution by a needle moving with different velocity till some draw ratio (Figure 1).

The experiment involves drawing a drop produced by a syringe and transforming the drop into the jet at different speeds. The most original element of this device is the system of lighting. The light beam from a halogen lamp (150 W) passes through optical fiber and is focused in the center of a drop by means of a microscope objective. In other words, a stretching jet itself plays the role of optical fiber. In addition, the backlighting was used to make clear boundaries of a jet/fiber. Videography started simultaneously with moving of a needle with a drop of solution. The resolution was 1920 × 1080 with a frequency of 60 shots per second. The objective used (produced by LOMO, St-Petersburg, Russia) provides the necessary clarity and depth of vision.

**Figure 2:**
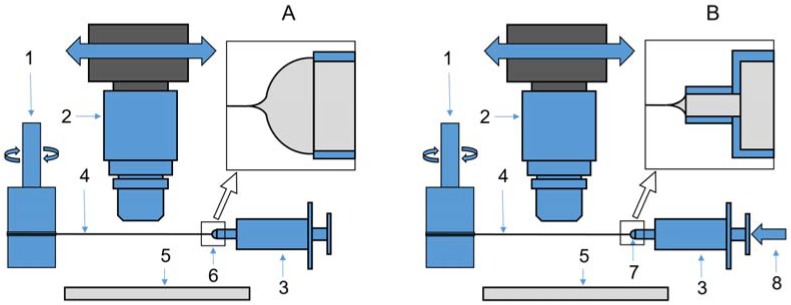
Experimental devices for fiber spinning with constant speed. Spinning is carried out from a solution drop (**A**) or syringe orifice (**B**). 1—rheometer with rotating cylinder which is used for measuring torque at fiber winding; 2—a camera moving along a fiber with a lens of high magnification; 3—syringe with a polymer solution; 4—monofilament under spinning; 5—back lightening; 6—drop of a solution; 7—syringe orifice; 8—constant-rate supply engine.

**Figure 3:**
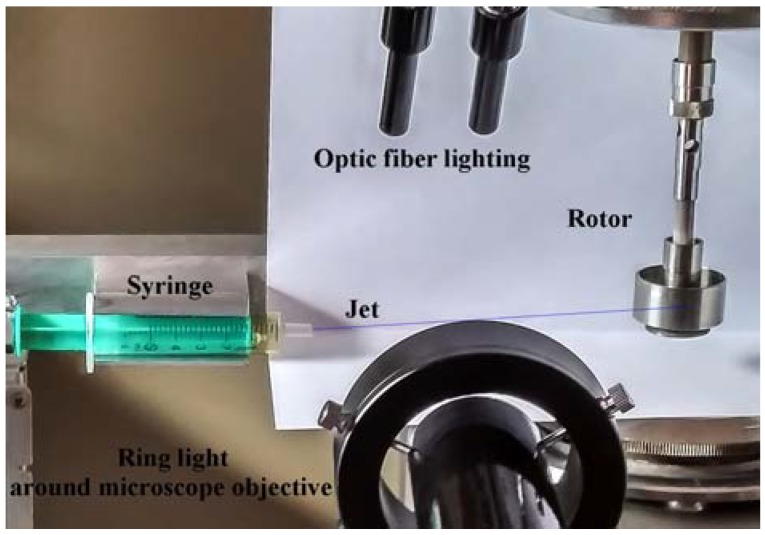
General view of the experimental setup for continuous mechanotropic spinning.

The second device is a rather complicated experimental setup modeling a real process of fiber spinning and winding of spun fiber in the continuous mode. The scheme is shown in Figure 2 and the real view is presented in Figure 3.

In this setup a fiber is extended and wound on the rotor of a Rheostress RS600 (Thermo Co., Karlsruhe, Germany) rotational rheometer. This allows us not only to realize a stable long-term (as long as we wish) spinning process with different and controlled strain rates, but also to measure the torque acting on the rheometer rotor and then recalculate it to the tensile stress stretching a jet (fiber). In these experiments we varied the rotation speed from 8 to 83 rpm and the average deformation rate from 0.1 to 1 s^−1^, providing a draw ratio from 50 to 500 (for a length of 20 cm). The spinning process consisted of flow of solution out of a spinneret channel with diameter of 500 µm and length-to-diameter ratio of 10:1 with subsequent filament winding on a rotor of rheometer.

All experiments were carried out with the constant flow output of 0.005 mL/min. The system of visualization was arranged on the base of the camera Touptek XFCAM1080PHD (ToupTek Co., Zhejiang, China) with LOMO Co. (St. Petersburg, Russia) microscope with magnification of 10× and the aperture of 0.25 or 4× and aperture of 0.11 for the wide-angle vision. As a result, images with resolution of 1 (3.3 for 4× magnification) µm/pixel having good clarity and depth of focus were obtained.

## 3. Results and Discussion

### 3.1. Rheological Properties of Samples

**Figure 4:**
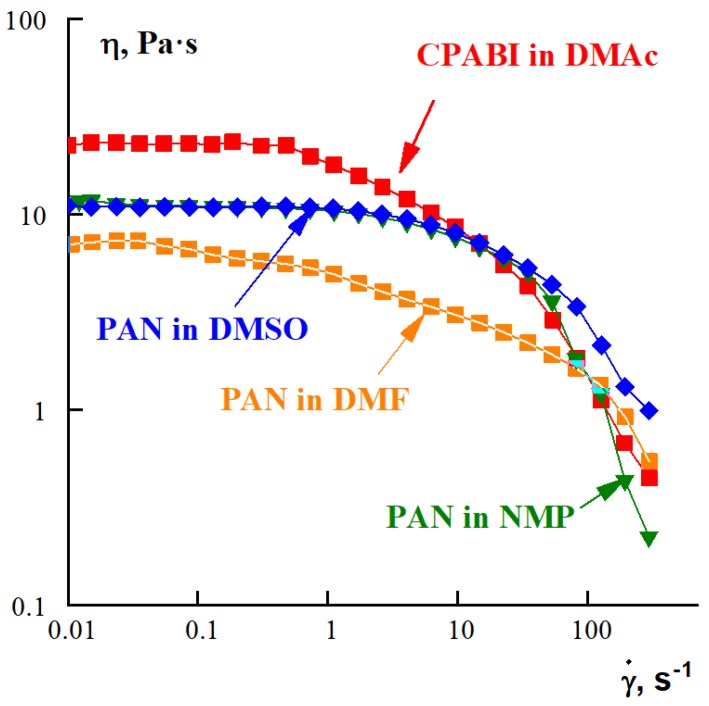
Viscous properties (non-Newtonian flow curves) of CPABI and PAN solutions in different solvents (shown at the curves).

The stable spinning process can be realized only from solutions of definite rheological properties. Therefore, it was necessary to characterize the rheological properties of the samples under study. Flow curves of several samples are shown in Figure 4. It is seen that all curves are close to each other forming a rather narrow band. These are quite typical viscous properties of polymer solutions used for fiber spinning.

**Figure 5:**
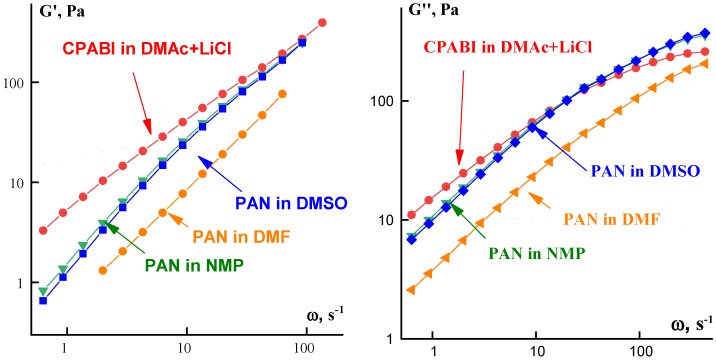
Frequency dependences of the storage (**left**) and the loss moduli (**right**) for PAN solutions in different solvents and the CPABI solution (shown at the curves).

Polymer solutions are viscoelastic media, and this is important for a possibility of extension. This is a principal point for spinning of polymer solutions because just elasticity provides a possibility of large deformations and absence of the Plateau–Rayleigh instability in jets of concentrated polymer solutions [75]. Relevant experimental data are presented in Figure 5.

These curves are quite typical for viscoelastic liquids in the frequency domain transient from the terminal zone to the viscoelasticity plateau. The slope of the $G^{\prime }\left(\omega \right)$ dependencies is close to 1.5, which reflects a rather wide relaxation spectrum. The slope of the $G^{\prime \prime }\left(\omega \right)$ dependencies is equal to 1 at low frequencies and gradually decreases with increasing frequency. The intercept of the $G^{\prime }\left(\omega \right)$ and $G^{\prime \prime }\left(\omega \right)$ curves—the crossover point—lies at frequencies of the order of 50±10 s^−1^ that corresponds to the characteristic relaxation time of the order of 0.02 s.

### 3.2. Fiber Spinning

The aim of the experiments was to provide support for a principal possibility of obtaining fibers from polymer solutions by a method of mechanotropic spinning for polymers of two types. The first one is a typical fiber-forming polymer widely used in the textile industry (PAN). The second one is a rigid-chain polymer (CPABI) of special interest for production of high performance fibers used as reinforcing agents in composite materials. 

**Figure 6:**
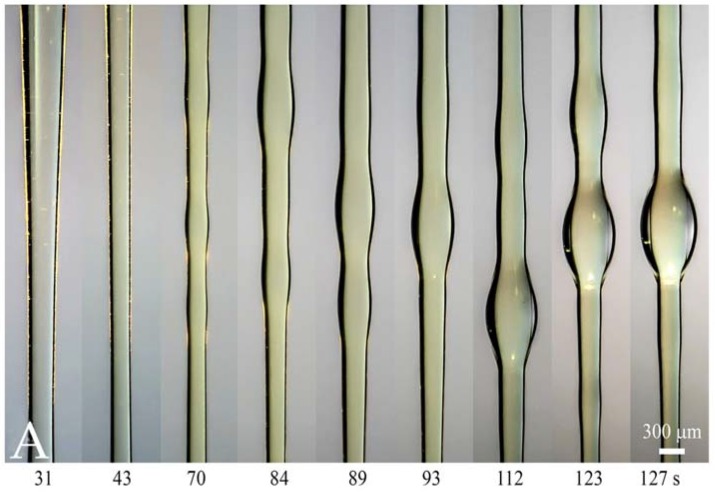

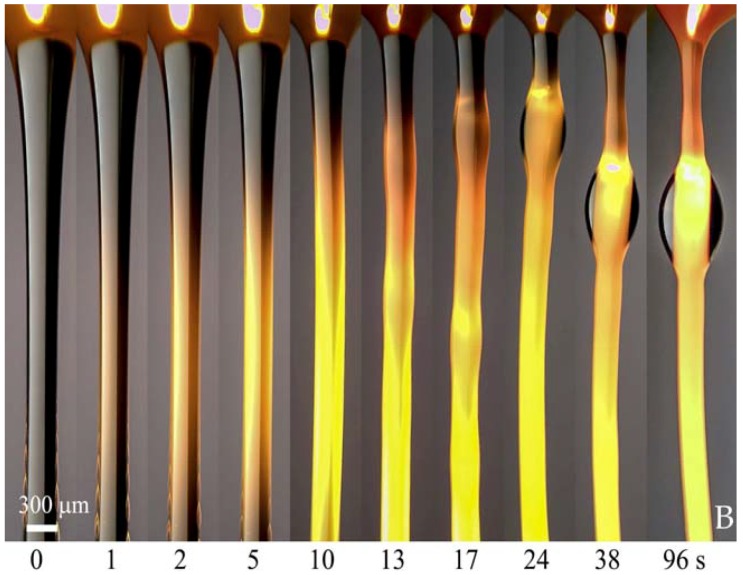
Phase separation at constant draw ratio for 7 wt. % of CPABI in DMAc+3% LiCl (**A**) and PAN solutions in DMSO (**B**). Appearing wavy jet shape and glow reflect inhomogeneity obliged to phase separation.

Figure 6A demonstrates the typical time evolution of the phase separation at the constant draw ratio for solution of CPABI in DMAc +3% of LiCl. A quite similar picture was observed for the PAN solutions (Figure 6B).

**Figure 7:**
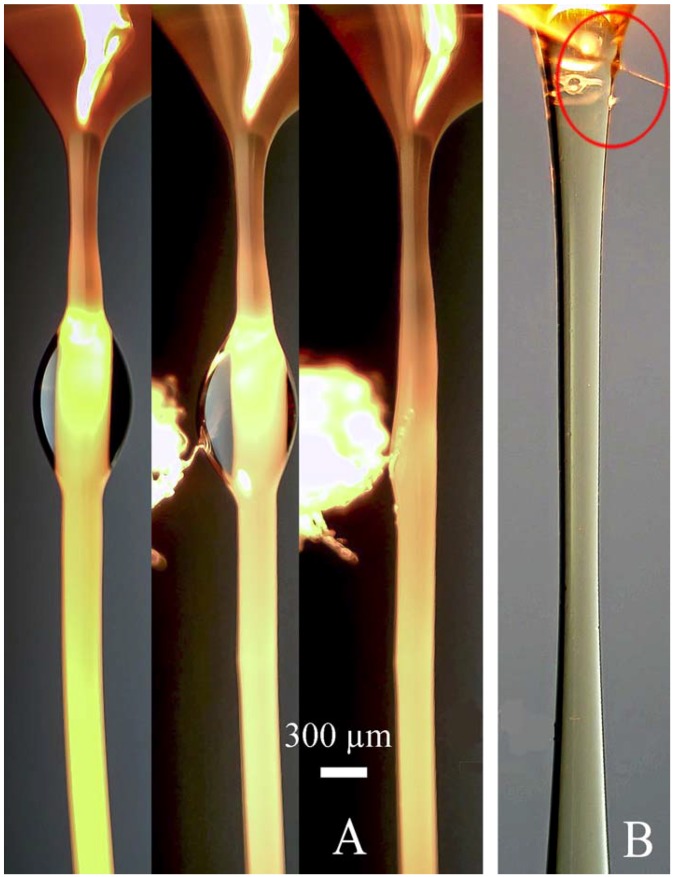
Removal of unit separated droplets from surface of the PAN (**A**) and from the Taylor cone in CPABI fiber spinning (**B**).

The separated droplets (but not beads inside the structure of a fiber) can be easily removed from an oriented fiber by a needle with the cotton lint (as shown in Figure 7).

The formation of separate solvent droplets is the final stage of the phase separation, which takes place initially through the whole section of a fiber. As said above, this phenomenon has been demonstrated through application of the special technique of passing a light beam along a fiber playing the role of the optical waveguide (Figure 1). Phase separation leads to appearance of inhomogeneity in the structure of the cross-section of a fiber. This is reflected by the local glow due to light scattering of a ray in these sections. This glow is seen in some screen-shots presented above. 

A possibility of quick removal of a solvent is followed from comparison of the intrinsic time scales of the stretching process t* and diffusion *t_D_*. The time of diffusion is estimated as $t_{D}=\frac{r^{2}}{D}$, where *r* is a characteristic size and *D* is the coefficient of diffusion. In our case, the radius of a fiber is of the order of 5 µm, and the coefficient of diffusion in the system PAN-DMSO lies in range of 10^−5^–10^−6^ sm^2^/s (according to the known experimental data [76,77,78]). Therefore, *t_D_* is the value of the order of 0.01–0.10 s. The characteristic time of the process, t* can be estimated as $t*=\frac{L}{V}$ where *L* is the distance between the spinneret and the winding roll, and *V* is the speed of winding. For the used conditions t* lies between 1 and 10 s. It is seen that *t_D_* << t* and the solvent has enough time to release the jet/fiber. 

**Figure 8:**
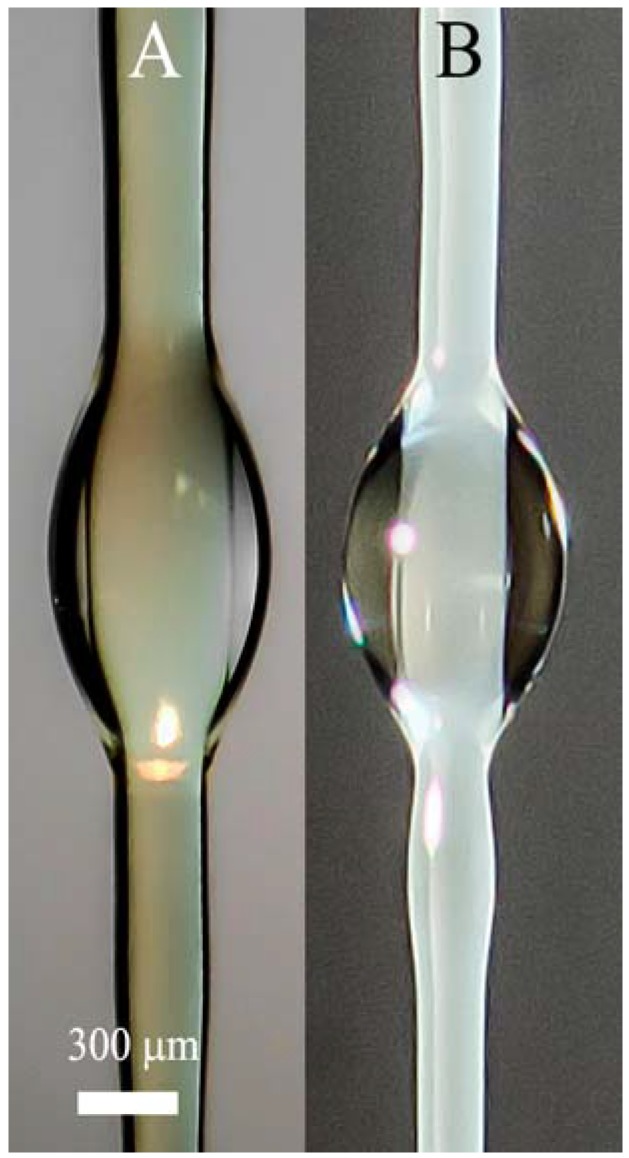
Enlarged images of solvent droplets on fiber surfaces for CPABI in DMAc solution (**A**) and PAN in DMSO solution (**B**).

Solvent under extension is squeezed out onto the surface, forming a thin covering layer. Then instability appeared due to the action of surface forces leads to the transformation of this layer into wave flow (as firstly was described in [75]). These waves can slowly disintegrate into separate droplets (as in Figure 8), or move along the surface to the Taylor cone as seen in screen-shots in Figure 7B. The formation of solvent droplets is governed by superposition of inertial and capillary forces as well as the kinetics of solvent diffusion from volume to fiber surface. Similar pictures of formation of separate solvent droplets have been first demonstrated in our earlier publication [71], and are now confirmed for different polymers and other conditions of spinning. Therefore, we can conclude that phase separation is a general type of polymer solution behavior at high enough deformation rates.

**Figure 9:**
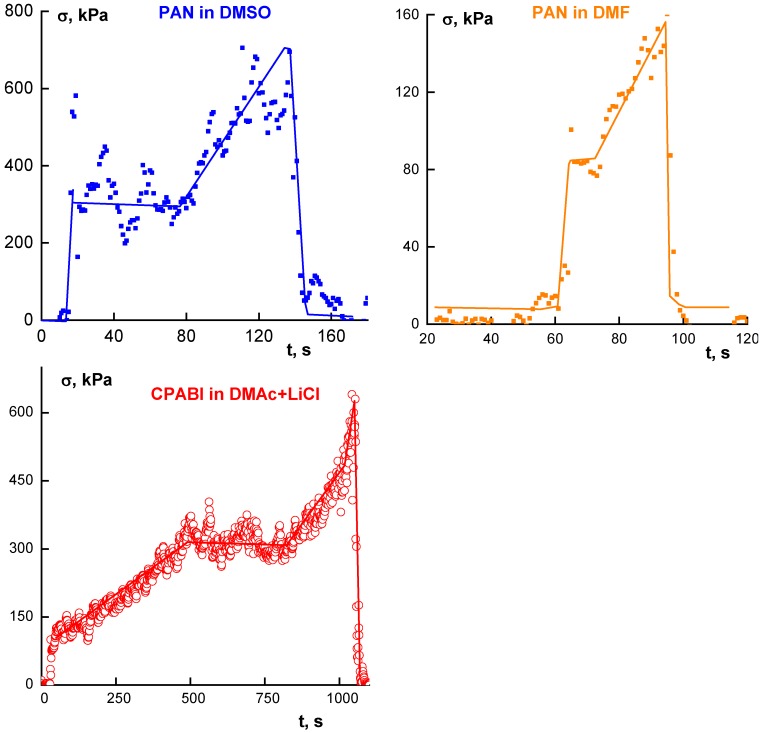
Stress development in extension of drops of PAN solutions in different solvents (upper rank) and of CPABI solution in DMAc + LiCl.

In spinning from a drop, the phase separation is accompanied by a rather interesting picture of stress evolution. Stresses were calculated from the torque measured in winding a fiber on the cylinder of the rheometer. Some characteristic pictures are shown in Figure 9 for PAN solutions in different solvents and for CPABI.

There are some common features in these dependences, separated on different stages of the stress evolution. An initial increase in force is required to stretch a solution, the plateau corresponds to some equilibrium state. Presumably, plateau appears because of destroying the solvation in a jet, i.e., transition from solution to mechanical mixture of the gel-like polymer and solvent. At acting the tensile force on such “soft matter” the stress could be constant. The other reason for the plateau existing can be stipulated by multi-necking that takes place on the final stages of mechanotropic process. Then, almost complete removal of a solvent from the jet/fiber body accompanied by increasing rigidity of a filament causes an increase of tensile stress. 

The breakup happens just at this stage and is seen as the abrupt fall of force at the exhaustion of its durability. It is worth mentioning that the limiting stress strongly depends on the nature of a solvent. This effect (dependence of durability on the nature of a solvent used for fiber spinning) was described for many polymers [76]. 

**Figure 10:**
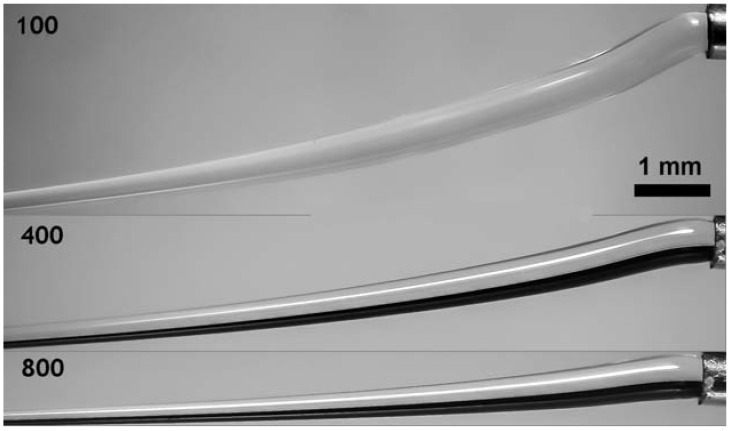
Extension of jets in PAN solution spinning at different rates (indicated in photos).

The results of the model experiments gave us the basis for arranging the stable process of spinning. Figure 10 shows the variation of the fiber diameter for different deformation rates.

**Figure 11:**
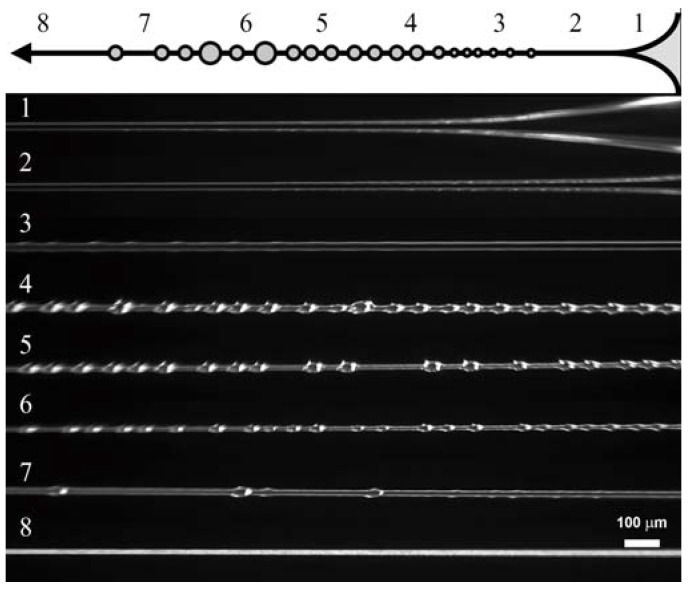
Continuous spinning of fibers from 7% solution of CPABI. Frames correspond to different distance from the spinneret.

**Figure 12:**
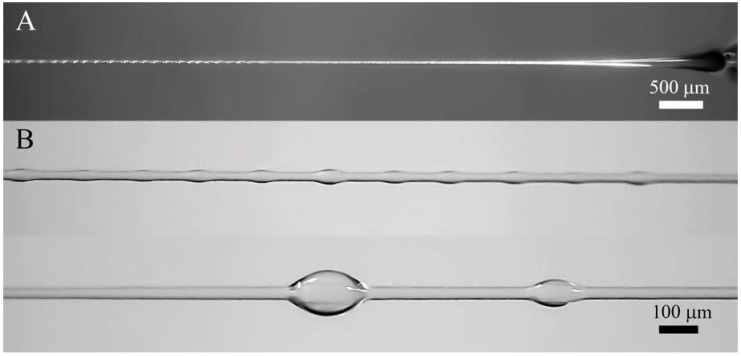
Images of continuous spinning of PAN fibers at different magnification: (**A**)—general view; (**B**)—enlarged images of the jet section.

The process of phase separation is shown for two examples presented below (Figure 11) which illustrates the kinetics of droplet formation along the Armos fiber at different distances from the spinneret, and is the scanning of the spinning process. Droplet formation during PAN spinning from its solution in DMSO is shown in Figure 12. The droplets become clearly detectable upon magnification of the image.

**Figure 13:**
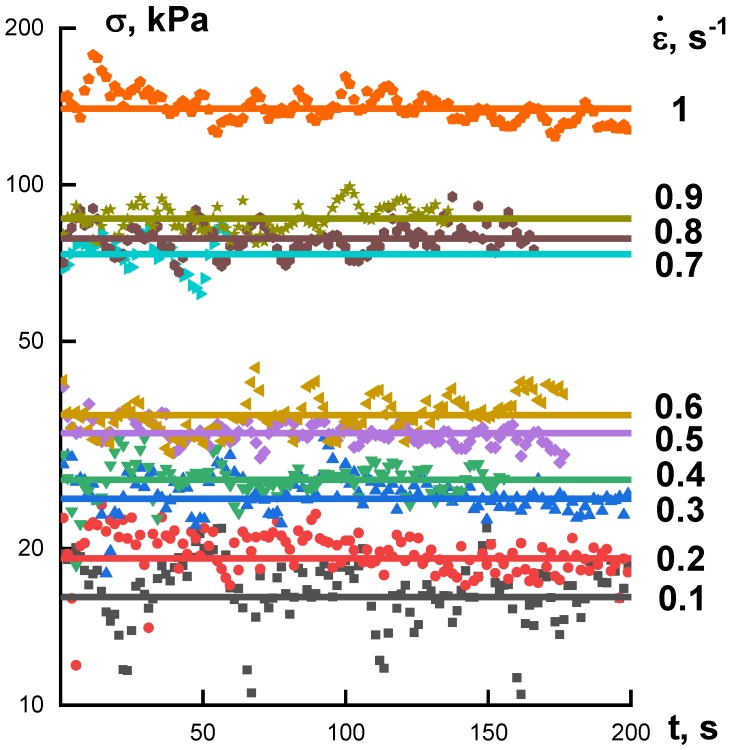
Stresses during stationary stage of the continuous spinning of PAN from DMSO solutions.

A stable process of spinning is determined by the rheological properties of a solution. These properties are responsible for stresses developed in extension, and the limiting stress is related to the strength of a fiber, as was shown in Figure 9. Therefore, the realized rate of winding depends on the solvent nature and the permissible stress. Figure 13 shows the results of measuring tensile stresses in spinning.

**Figure 14:**
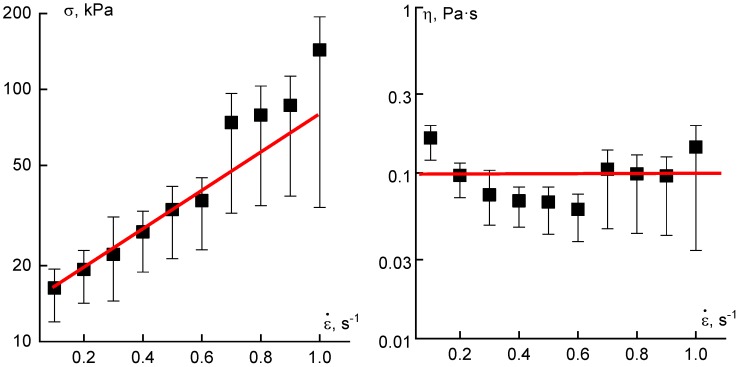
Dependences of the average stress (**left**) and calculated apparent elongation viscosity (**right**) on the average deformation rate in continuous spinning process of PAN solution.

In experiments with extension up to the given length (described above), stresses increase in time in a way that can be related to the transient character of the process of phase separation. In continuous spinning, tensile stress is constant (on average) though frequent oscillations exist. This constant stress increases along with growth of the rate of deformation (i.e., speed of winding). Meanwhile, the apparent elongation viscosity (calculated as the ratio of the tensile stress to the deformation rate) remains practically constant (Figure 14). The jet profile can change along the path of drawing. Therefore, stress and deformation rate are not constant along a fiber, but the apparent extension viscosity might be constant. Nevertheless, Figure 14 presents only a qualitative picture, though a useful one for understanding parameters governing the spinning process.

**Figure 15:**
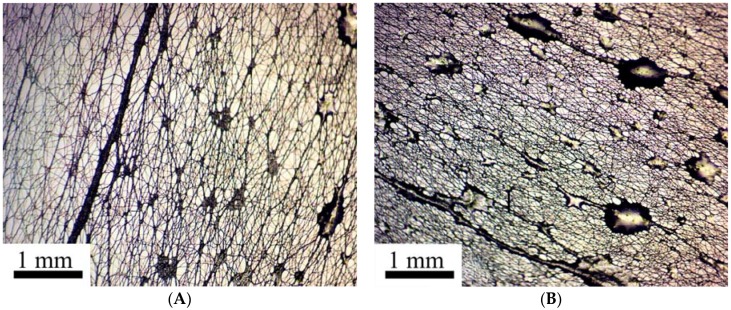
Photos of PAN—web in 5 (**A**) and 10 min (**B**) after electrospinning from DMSO solution.

Coming back to comparison of mechanotropic and electrospinning, it is reasonable to indicate that continuous fibers can be also obtained at extension of aqueous solution jets in electric field. A good example on continuous fiber spinning in electric field of poly(*N*-isopropyl acrylamide) is demonstrated in [79]. Water is an ecologically clean solvent with relatively low boiling point. That is why a majority of electrospun fibers are prepared from water soluble polymers. For electrospinning of PAN and CPABI, soluble in solvents having high vapor pressure and high boiling temperature, i.e., DMAc, DMSO, it is possible to observe the solvent drops on fibers surface without evaporation proceeding in time (Figure 15). 

This means that depending on the phase state of a solution and a type of the solvent, both mechanisms (phase separation and solvent evaporation) may coexist [80].

## 4. Conclusions

The principle possibility of a novel technology of fiber spinning based on the effect of phase separation under strong tension stresses is proposed and examined for two different polymers. Phase separation happens across the section of the solution jet that can be observed by appearance of turbidity inducing strong light scattering. Then a solvent forms a thin layer (cover) on the oriented fiber. The further fate of this cover is determined by superposition of surface tension and mechanical forces. The most impressive consequence of the phase separation is the formation of the solvent drops located on the fiber surface but not entering backward. Stress evolution in extension as well as elongation viscosity was measured. The model experiments have determined the conditions at which the effect under discussion allowed for realizing a spinning process in the stable long-term mode with different winding speeds.

The analysis of the physics behind the advanced technology showed that the phase separation in extension of polymer solutions proceeds due to changing in the character of polymer-solvent interaction as a result of extension of macromolecular chains and the coil-to-aligned chain transition. This leads to the shift of the spinodal of a polymer-solvent system and finally to demixing the solution.

## Figures and Tables

**Figure A1:**
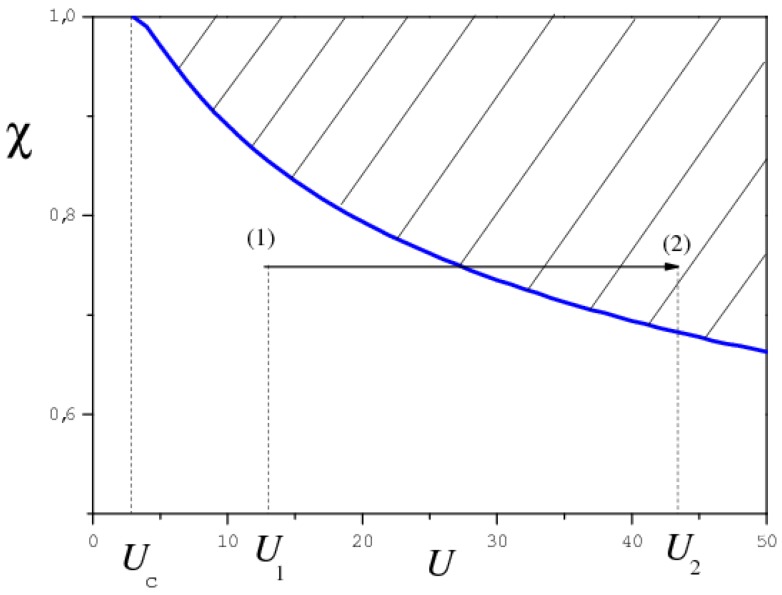
The instability area (shaded) in the coordinates (U,χ). Point (1) corresponds to the initial extension rate close to the die in the fiber spinning process and (2) is the final point where the external tension force is applied.

**Figure A2:**
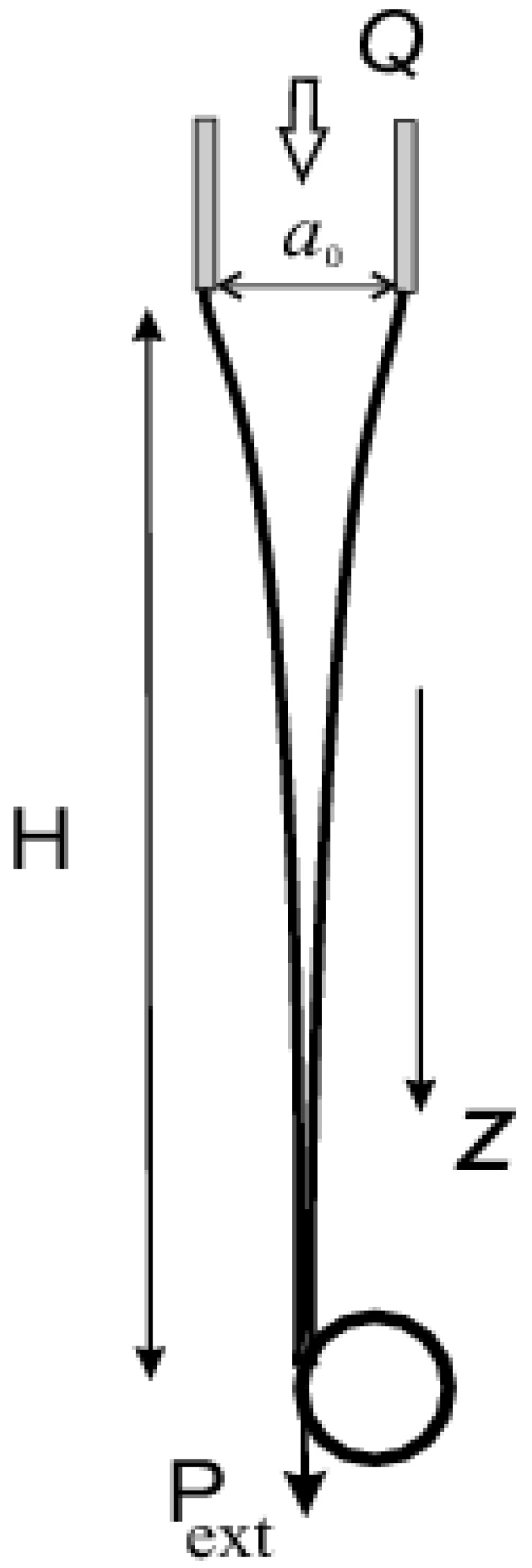
Schematic picture for the fiber spinning.

## Appendix A

### Theoretical Considerations

The theoretical background of the proposed novel technology of fiber spinning is the analysis of the physics of phase separation under deformation. The theory of phase demixing in dilute polymer solutions undergoing uniaxial stretching was recently proposed [81,82]. Separation of the components in a solution in this theory is related to the chain’s orientation. In the oriented state, the steric repulsion between the polymer segments decreases, whereas the attractive part of interactions only weakly depends on the orientation. Therefore, balance between the attractive and repulsive forces moves towards attraction, and the turbidity point, reflecting the phase separation, moves along a fiber depending on the elongation rate. The proposed physical approach is applicable for concentrated solutions also. Some complications appear at high concentrations where entanglements between polymer chains become important; where entanglements between polymer chains become important; however, these effects will not be considered in this paper. Meanwhile the nature of phase demixing in the entangled polymer solutions including solutions of rod-like polymers remains the same and is connected with the orientation of the polymer chains or their parts between junctions of entanglements.

Below, behavior of concentrated polymer solutions without entanglements undergoing uniaxial elongational flow (along z-axis) with deformation rate $\dot{\varepsilon }$ will be discussed.

The polymer chains have diameter d. The Kuhn segment length l and contour length are $L=Nl_{1}$ where $l_{1}$ is the length of the monomer units ($l_{1}\approx l$) and N is the polymerization degree. In equilibrium, a polymer coil is characterized by the size $R_{0}={\left(Ll\right)}^{1/2}$ whereas in the stretched state their size $R_{z}$ (along z-axis) is governed by the hydrodynamic friction force stretching a chain. Orientation of the chain segments in the stretched state is characterized by the order parameter $s=R_{z}/L=\langle \cos\theta \rangle$, where θ is the angle between a chain segment and z-axis. The angular brackets imply averaging with respect to the chain conformations. A size of the stretched coils $R_{z}$ is found from the balance between the work of the friction forces stretching a chain, $F_{ext}$, and the chain’s elastic energy $F_{el}$. The elastic energy for a wormlike chain is written as $F_{el}\sim \frac{k_{B}TL}{2l}\tilde{F}\left(R_{z}/L\right)$ [82] where $\tilde{F}$ is implicitly defined by the following equations including the auxiliary parameter A: $\tilde{F}=A\coth A-1$, $R_{z}/L=s=\coth A-1/A$.

In concentrated polymer solutions, the hydrodynamic interactions are screened. So the friction force per monomer unit is $f_{n}=\zeta \upsilon_{n}$ where ζ is the friction coefficient and $\upsilon_{n}=\dot{\varepsilon }sl_{1}\left(n-N/2\right)$ is the relative velocity between the monomer with number n and the flow, n=0⋯N. The average tension force acting on the chain is created by the friction

(A1) $$h\sim \frac{2}{N}\int_{N/2}^{N}dm\int_{m}^{N}f_{n}dn=\frac{1}{12}\zeta \dot{\varepsilon }sl_{1}N^{2}$$

Hence the work done to stretch a chain is given by

(A2) $$F_{ext}=-\int_{0}^{R_{z}}hdR_{z}=-\frac{1}{24}\zeta \dot{\varepsilon }s^{2}l_{1}^{2}N^{3}$$

Here we used the relation $R_{z}=Nl_{1}s$. This work is the quadratic function of the order parameter $F_{ext}\propto s^{2}$ and differs from the work of stretching of an isolated coil in the dilute solution where $F_{ext}\propto s^{3}$ [81,82]. The total energy of the stretched chain is written as

(A3) $$F=F_{el}+F_{ext}\approx \frac{k_{B}TL}{2l}\left[As-Us^{2}\right],\;U=\frac{1}{12}\frac{\left(\zeta /l_{1}^{}\right)\dot{\varepsilon }lL^{2}}{k_{B}T}$$

Here $\zeta /l_{1}^{}$ is the friction per unit chain length and parameter U increases linearly with the draw rate. Minimization of the free energy (3) allows us to find the stretched coil size and the order parameter as a function of the parameter U, s=s(U). The analysis shows that a chain starts to stretch when $U>U_{c}=3$ or at $\dot{\varepsilon }>{\dot{\varepsilon }}^{*}=\frac{1}{2\tau_{R}}$ where $\tau_{R}=\frac{1}{18}\frac{\left(\zeta /l_{1}^{}\right)lL^{2}}{k_{B}T}$ is the Rouse relaxation time. The coil-to- stretched chain transition in this case is continuous and for $U>U_{c}$ the order parameter s(U) increases monotonically.

As has been mentioned above, orientation of chains happens as a result of reduction of the steric repulsion between the polymer segments, and this effect increases with increases in the elongation rate $\dot{\varepsilon }$ or the parameter $U\propto \dot{\varepsilon }$. As far as attraction between the chain segments slightly depends on the orientation, the phase separation between polymer and solvent can occurs once the osmotic modulus $\kappa =\frac{1}{k_{B}T}\frac{\partial \Pi }{\partial c}$ becomes negative. Here the osmotic pressure Π in the polymer solution is a function of concentration c of the monomer units. The osmotic pressure involves the ideal gas term and contribution due to interactions:

(A4) $$\Pi =k_{B}Tc\left(\frac{1}{N}+\frac{l_{1}}{d}\phi k\right)$$

where k=I(s)−χ(T) and $\phi =\frac{\pi }{4}d^{2}l_{1}c$ is volume fraction of polymer in the solution. Here the function $I\left(s\right)=\frac{4}{\pi }\langle \sin\beta \rangle$ accounts for anisotropy of the steric repulsion between polymer segments (β is the angle between two randomly chosen segments), and the parameter χ(T) characterizes non-steric interactions between different segments and between the segments and the solvent, at that $\chi (T_{\Theta })=1$ where $T_{\Theta }$ is Θ—temperature. For 1−s~1 the function is $I\left(s\right)\sim \sqrt{1-s}$. The osmotic modulus of the polymer solution is written as $\kappa =\frac{1}{N}+\frac{2l_{1}}{d}\phi k$. When the modulus is negative (κ<0), the polymer solution separates into polymer and solvent phases. The minimal concentration at which the phase separation occurs is estimated as $\phi_{dem}∼\frac{d}{Nl_{1}\left|k\right|}$. It is inversely proportional to the M_W_ of the chains. At $\phi \sim \phi_{dem}$, the transition to the instability regime (the turbidity point) is found from equation χ(T)~I(s(U)), presented in Figure A1. The polymer volume fraction $\phi_{c}$ in the polymer phase after the phase separation occurs is obtained from the equilibrium condition, with the solvent phase implying Π~0 and yields $\phi_{c}\sim \left|k\right|/\chi$.

In fiber spinning, polymer solution goes from the die of the radius $a_{0}$ with the flow rate Q and then stretched by the external force $P_{ext}$ applied on a distance H from the die (Figure A2).

The radius of the filament in the stationary regime is a function of the distance z from the die, a=a(z). The velocity and the extension rate inside the filaments are $\upsilon (z)=\frac{Q}{\pi a^{2}}$ and $\dot{\varepsilon }(z)=-\frac{2Q}{\pi a^{3}}\frac{da}{dz}$. The dynamics of the filaments are described by the momentum equation [78]

(A5) $$\frac{d}{dz}\left[a^{2}\left(\rho \upsilon^{2}-N_{1}\right)\right]+\gamma a^{2}\frac{dC}{dz}=0$$

where $N_{1}=\sigma_{zz}-\sigma_{rr}$ is the normal stress (r is the radial coordinate), ρ is the density, γ is the surface tension and C is the total curvature of the free surface. Here, gravity forces are neglected. In the intermediate stretching regime where the size of the polymer coils $R_{z}$ obeys the inequality $R_{0}<R_{z}<L$, the Oldroid-B model can be used [83]

(A6) $$\tau_{R}\upsilon \frac{d\sigma_{zz}}{dz}-2\tau_{R}\frac{d\upsilon }{dz}\sigma_{zz}+\sigma_{zz}\approx 2\eta \frac{d\upsilon }{dz}$$

(A7) $$\tau_{R}\upsilon \frac{d\sigma_{rr}}{dz}+\tau_{R}\frac{d\upsilon }{dz}\sigma_{rr}+\sigma_{rr}\approx -\eta \frac{d\upsilon }{dz}$$

Here η is the viscosity of the polymer solution. Below, the case when the normal stress in the polymer solution exceeds the elastic modulus $G\approx \eta /\tau_{R}$ will be considered. Then $N_{1}\sim \sigma_{zz}$. If the viscoelastic forces in the system are dominant Equation (A5) is simplified to $\frac{d}{dz}\left(a^{2}N_{1}\right)\approx 0$, Therefore $N_{1}\approx P_{ext}/\left(\pi a^{2}\right)$ and Equation (A6) can be approximated as

(A8) $$\tau_{R}\upsilon \frac{dN_{1}}{dz}-2\tau_{R}\frac{d\upsilon }{dz}N_{1}+N_{1}\approx 0$$

After elimination of $N_{1}$ and υ from (A8), the equation for the filament profile can be achieved:$\frac{2Q\tau_{R}}{\pi a^{3}}\frac{da}{dz}+1\approx 0$. Then the radius yields as $a=a_{0}{\left(1+\frac{\pi a_{0}^{2}z}{Q\tau_{R}}\right)}^{-1/2}$. It asymptotically decreases as $a\propto z^{-1/2}$. This scaling is connected with unfolding of the polymer coils.

In the thinning filament, $\frac{da}{dz}<0$, the extension rate increases, and therefore under some processing conditions the system could pass through the spinodal (see Figure 13). This transition is accompanied by phase demixing and the solvent releases from the polymer phase. Subsequent behavior depends on the demixing kinetics occurring in non-homogeneous extension regime. Some aspects of these kinetics for dilute solutions have been considered in [82]. However, the situation for concentrated solutions needs more detailed analysis.
